# Pediatric cystic lymphangioma of the retroperitoneum: A case report and review of the literature

**DOI:** 10.1097/MD.0000000000020827

**Published:** 2020-07-10

**Authors:** Fabio Poroes, David Petermann, Snezana Andrejevic-Blant, Ismail Labgaa, Luca Di Mare

**Affiliations:** a Department of General and Visceral Surgery, EHC Hospital, Morges; b Unilabs-Cypa, Department of Pathology, Lausanne; c Department of Visceral Surgery, Lausanne University Hospital CHUV, Lausanne, Switzerland.

**Keywords:** benign tumor, pediatric surgery, retroperitoneal, surgical resection

## Abstract

**Rationale:**

Cystic lymphangioma (CL) is a rare benign tumor resulting from a failure of the lymphatic system development. It may occur at any age but it is more frequent during childhood. Its clinical presentation and location are various but abdominal CL are uncommon. Among those, mesenteric presentation is the most frequent form whereas CL of the retroperitoneum are particularly rare.

**Patient concerns:**

Herein, we report the case of a 17-years-old patient with no medical history who presented with right-upper quadrant (RUQ) pain, but no other symptom. Physical examination showed tenderness of the RUQ without distension. Lab tests were unremarkable.

**Diagnosis:**

Abdominal computed tomography (CT) highlighted a retroperitoneal cystic mass potentially infiltrating the mesenterium, raising suspicion of a CL of the retroperitoneum. Diagnosis of CL was confirmed by histological analyses.

**Intervention:**

Patient underwent an exploratory laparoscopy that infirmed infiltration of the mesenterium and allowed for resection.

**Outcomes:**

Postoperative course was uneventful and there is no evidence of recurrence after 14 months of follow-up.

**Lessons:**

Although CL essentially occur in children, pediatric retroperitoneal CL is a rare finding, with only 21 cases identified in the literature.

In summary, CL are benign tumors rarely located in the retroperitoneum. Despite performant imaging technologies, preoperative diagnosis is challenging. Whenever possible, laparoscopic resection should be the treatment of choice. Herein, we report the largest CL pediatric case laparoscopically resected, and the first review of the literature on the topic.

## Abstract 2

Supplemental Digital Content is available in the text

## 1 Introduction

Cystic lymphangioma (CL) is a rare benign tumor resulting from a failure in the development of the lymphatic system that can occur at any age but more typically during childhood.^[1]^ Craniofacial, cervical or axillary localisation are the most common locations. Intra-abdominal forms are rare. Retroperitoneal localization of the CL is particularly uncommon.^[2]^ The clinical presentation of CL is various, ranging from incidental finding of abdominal cyst to acute abdominal presentation.^[3,4]^ Preoperative diagnosis is challenging and differential diagnosis is extensive.^[5]^ The diagnosis of CL mainly relies on imaging with either ultrasound (US), computed tomography (CT) or magnetic resonance imaging (MRI). It should thereafter be confirmed by histology.^[6,7]^ Whenever possible, complete resection should be attempted. The risk of recurrence primarily depends on margins status.^[4]^ Herein, we report the largest CL pediatric case laparoscopically resected, and the first review of the literature on the topic.

## 2 Case presentation

**Figure 1:**
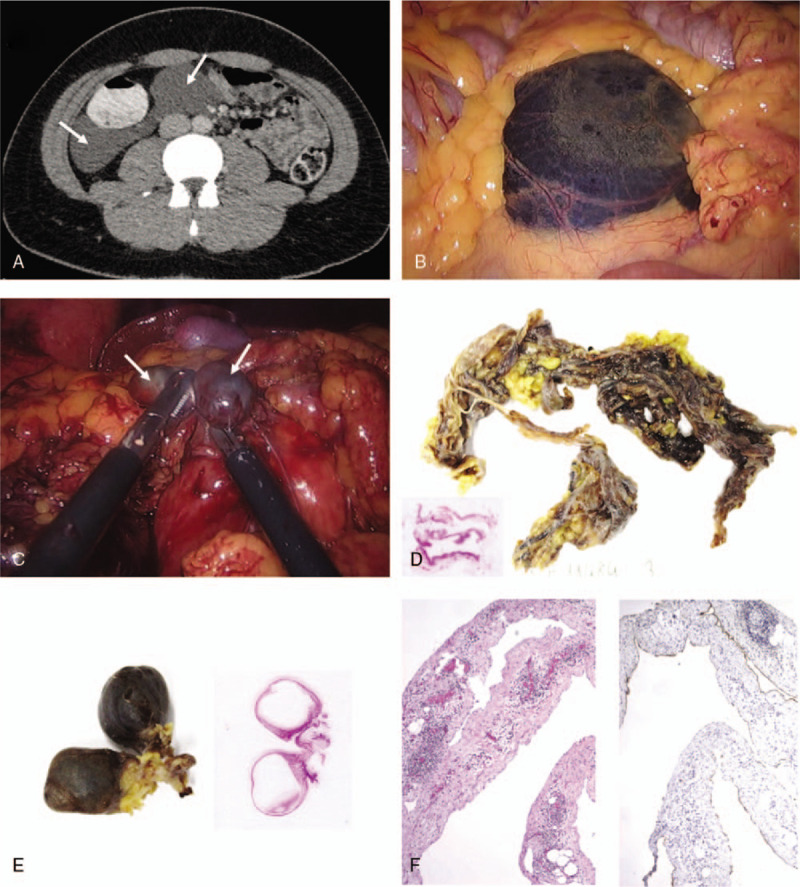
(A) Axial computed tomography (CT) image showing a well-defined retroperitoneal cystic mass (arrows) infiltring the mesenterium in relation to the right colic angle and right Morrison's pouch, measuring 14 cm of major axis. (B) Laparoscopic view of the main cystic adjacent to the last ileal loop. (C) Laparoscopic view of two smaller multi-loculated cysts (arrows) encapsulated within the main cyst. (D) Macroscopy and histology of the main cyst, with hematoxylin and eosin (H&E) staining. (E) Macroscopy and histology of the smaller multi-loculated cysts, with hematoxylin and eosin (H&E) staining. (F) Histology of the main cyst (4×), with H&E (left panel) and D2-40 (right panel) stainings.

A 17-year-old boy with no medical history presented with right-upper quadrant (RUQ) pain, but no other symptom. Physical examination showed tenderness of the RUQ but no rebound. Lab tests were unremarkable. An abdominal ultrasound revealed a right flank fluid collection of unknown etiology. An abdominal CT showed a retroperitoneal cystic mass infiltring the mesenterium near the right colic angle and right Morrison's pouch. The lesion measured 14 cm and raised the suspicion of a CL (Fig. 1A).

Patient underwent an exploratory laparoscopy that showed a large cystic mass close to caecum (Fig. 1B). Surgical exploration revealed a multiloculated cyst encapsulating other cysts of smaller sizes (Fig. 1C), and extending to the hepatic colonic angle without infiltrating the mesentery. The main cyst was filled with clear fluid which was aspirated and sent for cytological analysis. The tumor was resected almost entirely, only leaving a small portion of the capsule which was strongly adherent to the duodenum, precluding a safe complete resection. Postoperative course was uneventful and the patient was discharged on postoperative day 2. No sign of recurrence has been reported after 14 months of follow-up.

Macroscopically, specimens showed a bilobar cystic lesion containing hemorrhagic material measuring 3.0 × 1.2 × 1.0 cm and a thin fibrotic fragment of 23.0 × 2.0 × 1.0 cm.

Histological analyses of the resected cysts showed macrocystic cavities, containing thin fibrous tissue and lymphocytic infiltrate (Fig. 1D and E), boarded by a single layer of endothelial cells expressing D2-40 (Fig. 1F). These findings confirmed the diagnosis of CL. No malignant cells were detected by cytological analyses in the cystic fluid.

## 3 Discussion

Herein, we reported a rare case of symptomatic CL located in the retroperitoneum of a young patient successfully treated by surgery with laparoscopic resection.

CL is a rare benign tumor resulting from a failure in the development of the lymphatic system.^[1]^ Two theories exist about its origin. The first one relies on a malformation due to a lack of connection between abdominal lymphatic chains and venous system.^[2]^ The second one suggests an origin acquired due to inflammation, trauma or degeneration.^[5]^ The histological types of lymphangiomas are divided into cystic, capillary, and cavernous patterns. Retroperitoneal lymphangioma mostly shows cystic type.^[5]^

CL is most frequently located in the subcutaneous area of the cervix (∼75%) or axilla (∼20%).^[6]^ Intra-abdominal forms are observed in only 5% of cases.^[2]^ Retroperitoneal localization of the CL is particularly rare. Abdominal CLs are most common in children and 90% of the cases are diagnosed before the end of second decay of life, which is consistent with our 17-years-old patient.^[2]^

The clinical manifestations of abdominal CL are various.^[3]^ It varies from incidental discovery of an abdominal cyst to acute abdominal presentation. The most common symptom is abdominal pain often related to tumor volume, which can be associated with a palpable mass.^[4]^ Complications such as intestinal obstruction, intracystic hemorrhage, infection, torsion, spontaneous rupture of the cyst or digestive hemorrhage can cause acute abdomen.^[4]^

Differential diagnoses of cystic retroperitoneal lymphangioma include retroperitoneal hematoma, abscess, duplication cysts, ovarian cysts, microcystic pancreatic adenoma, pancreatic pseudocysts, mucinous pancreatic neoplasms, branch-type intraductal papillary mucinous neoplasia, lymphangiosarcoma, cystic metastases (gastric/ovarian), undifferentiated sarcoma, cystic teratoma, cystic mesothelioma, and malignant mesenchymoma.^[5]^

There is no specific sign and diagnosis is usually guided by imaging. To establish the diagnosis, ultrasound is the typical initial exam. CL appears as a sharply marginated, unilocular or multilocular liquid tumor, often with scattered echoes.^[8]^ In our case, ultrasound detected an anechoic fluid collection, well-delineated, without calcification and without any sign of complications. Considering the non-specific ultrasound aspect of these lesions, CT provides additional information on the size, extent of the lesion and its relation with adjacent structures. CL typically shows a well-circumscribed homogeneous cyst with contrast-enhancing walls and septa.^[8]^ MRI helps to better define the nature of the cyst, exhibiting hypointensity in T1 images and increased intensity in T2 images.^[9]^

The diagnosis of CL can only be confirmed by histological analyses and is based on well-established criteria.^[6]^ Those include a well circumscribed cystic lesion with or without endothelial lining, a stroma characterized by meshwork of collagen and fibrous tissue and a wall containing focal aggregates of lymphoid tissue.^[10]^ Lymphatic vessel endothelial receptor-1, vascular endothelial growth factor-3, monoclonal antibody D2-40 and prox-1 are used as immunohistochemical markers in the diagnosis of lymphangioma.^[11]^

Surgery is the cornerstone of CL treatment. Decision-making must take into account the benign nature of the tumor, potential complications, the infrequent spontaneous regression of the cyst and the need for definitive diagnosis.^[6]^ Surgical excision, open or laparoscopic, is the gold-standard for abdominal CL. The excision must be complete to reduce the risk of recurrences, which varies from 7% after complete resection, to 50% after incomplete resection.^[4]^

Our review of the literature only identified 21 cases of pediatric CL of the retroperitoneum.^[12–26]^ Details of the reported cases are provided in Supplementary Table 1. Characteristics of these cases are summarized in Table 1. Briefly, a Pubmed search was performed using the following terms “Retroperitoneal”, “cystic” and “lymphangioma” (between January 1970 and January 2019). Each abstract was carefully reviewed by two separate authors (FP and IL). Inclusion criteria were (I) retroperitoneal CL, (II) age under 18 years, (III) full-text available, (IV) manuscript written in English.

**Table 1:**
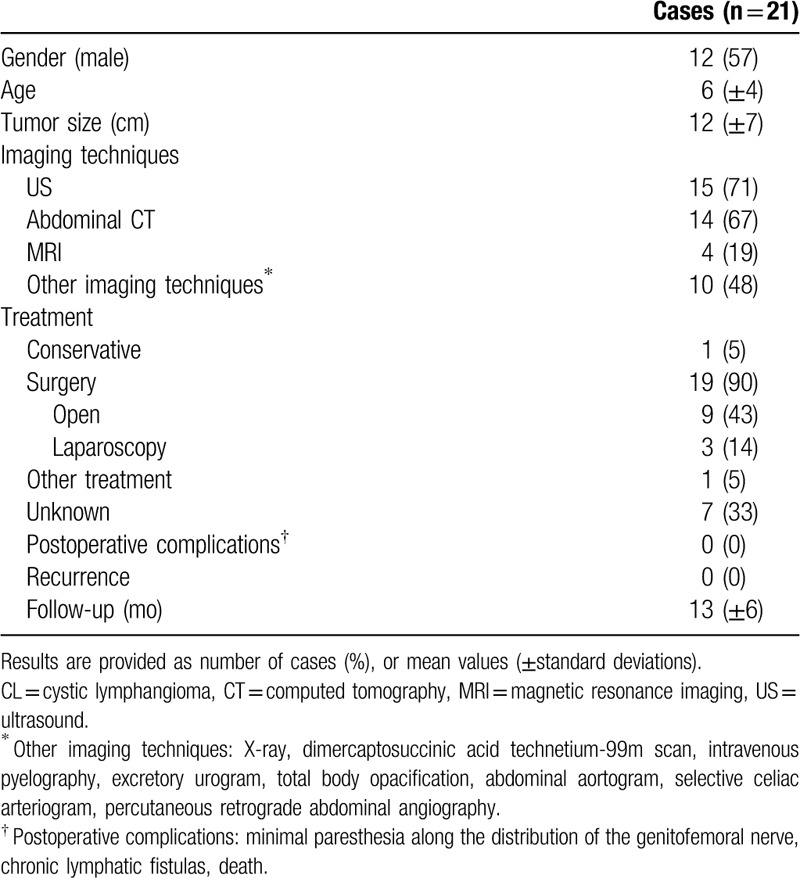
Characteristics of pediatric cases of retroperitoneal CL reported in the literature.

Overall, a majority of patients were male with an average age of 6 years. CL showed a mean size of 12 cm and were essentially diagnosed with ultrasound or CT. Surgery was performed in 90% of cases, mostly with open resection (43%). Outcomes were favorable with no reported postoperative complication and no recurrence.

In comparison to these similar cases, the present case reports the biggest retroperitoneal CL resected laparoscopically.

CL is a rare benign tumor resulting from a failure in the development of the lymphatic system affecting mainly children. Retroperitoneal localization is rare. The clinical presentation is various. Despite performant imaging technologies, preoperative diagnosis is challenging. Whenever possible, laparoscopic complete resection should be the treatment of choice.

## Author contributions

**Acquisition of data:** FP, SAB, IL, LDM

**Analysis and interpretation of data:** FP, DP, SAB, IL, LDM

**Critical revision of the manuscript for important intellectual content:** FP, DP, SAB, IL, LDM

**Drafting of the manuscript:** FP, IL

**Study concept and design:** FP, IL, LDM

## Supplementary Material

Supplemental Digital Content

## References

[1] Chaker K Sellami A Ouanes Y Retroperitoneal cystic lymphangioma in an adult: a case report. Urol Case Rep 2018;18:33–4. 29785367 10.1016/j.eucr.2018.02.019 PMC5958815

[2] Fattahi AS Maddah G Motamedolshariati M Chronic low back pain due to retroperitoneal cystic lymphangioma. Arch Bone Jt Surg 2014;2:72–4. 25207319 PMC4151444

[3] Celia A Breda G Laparoscopic Excision of a Retroperitoneal Cystic Lymphangioma: An Insidious Case [Internet]; 2007. Available at: https://home.liebertpub.com/lap [cité 26 févr 2019]. Disponible sur: https://www.liebertpub.com/doi/abs/10.1089/lap.2006.0167. 10.1089/lap.2006.0167 17570777

[4] Surlin V Georgescu E Dumitrescu C Retropancreatic cystic lymphangioma – considerations upon a case. Rom J Morphol Embryol Rev Roum Morphol Embryol 2011;52: Suppl: 493–6. 21424100

[5] Gachabayov M Kubachev K Abdullaev E A huge cystic retroperitoneal lymphangioma presenting with back pain. Case Rep Med [Internet] 2016;2016:1–3. 10.1155/2016/1618393 PMC5097799 27843456

[6] Bhavsar T Saeed-Vafa D Harbison S Retroperitoneal cystic lymphangioma in an adult: a case report and review of the literature. World J Gastrointest Pathophysiol 2010;1:171–6. 21607159 10.4291/wjgp.v1.i5.171 PMC3097960

[7] Kasza J Brody FJ Khambaty F Laparoscopic resection of a retroperitoneal cystic lymphangioma in an adult. Surg Laparosc Endosc Percutan Tech 2010;20:e114–6. 20551789 10.1097/SLE.0b013e3181db79a7

[8] Bonhomme A Broeders A Oyen RH Cystic lymphangioma of the retroperitoneum. Clin Radiol 2001;56:156–8. 11222077 10.1053/crad.2000.0162

[9] Ionescu C Ionescu M Dumitrascu T Retroperitoneal cystic lymphangioma in a patient with previous surgery for seminoma: a case report. Mædica 2012;7:180–2. PMC3557429 23399992

[10] Ozdemir H Kocakoc E Bozgeyik Z Recurrent retroperitoneal cystic lymphangioma. Yonsei Med J 2005;46:715–8. 16259073 10.3349/ymj.2005.46.5.715 PMC2810581

[11] Tripathi M Parshad S Karwasra RK Retroperitoneal lymphangioma in an adult: a case report of a rare clinical entity. Case Rep Surg [Internet] 2015;2015:1–4. 10.1155/2015/732531 PMC4381689 25866696

[12] Rekhi BM Esselstyn CB Jr Levy I Retroperitoneal cystic lymphangioma. Report of two cases and review of the literature. Cleve Clin Q 1972;39:125–8. 5084378 10.3949/ccjm.39.3.125

[13] Leonidas JC Brill PW Bhan I Cystic retroperitoneal lymphangioma in infants and children. Radiology 1978;127:203–8. 635183 10.1148/127.1.203

[14] Iyer R Eftekhari F Varma D Cystic retroperitoneal lymphangioma: CT, ultrasound and MR findings. Pediatr Radiol 1993;23:305–6. 8414761 10.1007/BF02010922

[15] Meyer T Stöhr G Post S Retroperitoneal lymphangioma presenting as a mesenteric cyst. Eur J Radiol 1995;21:143–4. 8850511 10.1016/0720-048x(95)00710-8

[16] Irvine AD Sweeney L Corbett JR Lymphangioma circumscriptum associated with paravesical cystic retroperitoneal lymphangioma. Br J Dermatol 1996;134:1135–7. 8763441

[17] Waldhausen JH Holterman MJ Tapper D Identification and surgical management of cystic retroperitoneal lymphangioma in children. Pediatr Surg Int 1996;11:283–5. 24057641 10.1007/BF00178441

[18] Freud E1 Farkash U Cassella R Childhood retroperitoneal lymphangioma presenting following minor trauma. Injury 1999;30:380–3. 10505137 10.1016/s0020-1383(99)00099-6

[19] Khetarpal R Halwai G Marwaha RK Retro-peritoneal cystic lymphangioma in association with fetal hydantoin syndrome. Indian J Pediatr 1999;66:294–7. 10798073 10.1007/BF02761223

[20] Shankar KR Roche CJ Carty HM Cystic retroperitoneal lymphangioma: treatment by image-guided percutaneous catheter drainage and sclerotherapy. Eur Radiol 2001;11:1021–3. 11419147 10.1007/s003300000669

[21] Rani DV Srilakshmi R Malathi S Unusual presentation of a retroperitoneal lymphangioma. Indian J Pediatr 2006;73:617–8. 16877857 10.1007/BF02759928

[22] Wildhaber BE Chardot C Coultre CL Total laparoscopic excision of retroperitoneal cystic lymphangioma. J Laparoendosc Adv Surg Tech 2006;16:530–3. 10.1089/lap.2006.16.530 17004884

[23] Wilson SR Bohrer S Losada R Retroperitoneal lymphangioma: an unusual location and presentation. J Pediatr Surg 2006;41:603–5. 16516648 10.1016/j.jpedsurg.2005.11.057

[24] Pratap A Tiwari A Sah BP Infected retroperitoneal cystic lymphangioma masquerading as psoas abscess. Urol Int 2008;80:325–7. discussion 328. 18480641 10.1159/000127351

[25] Singh RR Govindarajan KK Bowen C Retroperitoneal cystic lymphangioma: a rare presentation in childhood, treated laparoscopically. J Laparoendosc Adv Surg Tech A 2009;19:249–50. 19215217 10.1089/lap.2008.0234

[26] Gümüştaş OG Sanal M Güner O Retroperitoneal cystic lymphangioma: a diagnostic and surgical challenge. Case Rep Pediatr 2013;2013:1–3. 10.1155/2013/292053 PMC3600274 23533897

