# Supplementary material for: Pediatric cystic lymphangioma of the retroperitoneum: A case report and review of the literature
Source: Medicine (Baltimore). 2020 Jul 10;99(28):e20827. doi: 10.1097/MD.0000000000020827 (PMC7360192; doi:10.1097/MD.0000000000020827)
Supplement: Supplemental Digital Content [file medi-99-e20827-s001.docx]

**Supplementary Table 1: Details of reported pediatric cases of retroperitoneal CL**

| **Authors** | **Year** | **Gender** | **Age**  **(years)** | **Symptoms** | **Imaging technique** | **Size (cm)** | **Treatment** | **Excision** | **Follow-up (months)** |
| --- | --- | --- | --- | --- | --- | --- | --- | --- | --- |
| Rekhi et al. ^12^ | 1972 | F | 2 | Failure to gain weight, Loose stools. | X-ray, angiography | NA | OR | Partial | 6 |
|  |  | M | 4 | Pain, vomiting, fever | None | NA | OR | Partial | NA |
| Leonidas et al. ^13^ | 1978 | M | 2 months | Incidental finding | X-ray, Excretory urogram, Total body opacification | 5 | NA | C | NA |
|  |  | M | 3 months | Fever, Irritability, Poor feeding, Vomiting | Excretory urogram, Total body opacification CT | 5 | NA | NA | NA |
|  |  | M | 4 months | Abdominal mass | Excretory urogram, Total body opacification, US, CT | 6 | OR | Complete | NA |
|  |  | M | 2 | Abdominal pain, Irritability, anorexia, Vomiting | Excretory urogram, X-ray, Abdominal aortogram, Selective celiac arteriogram | 8 | NA | NA | NA |
| Iyer et al. ^14^ | 1993 | M | 7 | Pain, Abdominal mass | US, CT, MRI | 11 | NA | NA | NA |
| Meyer et al. ^15^ | 1995 | F | 16 | Pain, Anorexia, Constipation | US, CT | 12 | O | NA | NA |
| Irvine et al. ^16^ | 1996 | M | 6 | Haematuria | US, MRI | NP | C | NA | 18 |
| Waldhausen et al. ^17^ | 1996 | F | 9 | Pain | US,CT | 8 | NA | Complete | NA |
|  |  | F | 9 | Abdominal mass | US, CT | 29 | NA | Partial | NA |
| Freud et al. ^18^ | 1999 | M | 6 | Abdominal distention, pain | Intravenous pyelography | NA | OR | Complete | 6 |
| Khetarpal et al. ^19^ | 1999 | M | 1,5 | Constipation, abdominal distention | X-ray, US, CT | 20 | NA | Partial | NA |
| Shankar et al. ^20^ | 2001 | F | 4 | Pain, Fever, Vomiting | US, 99mTc (DMSA) scan, CT | NA | AS | Not applicable | 12 |
| Rani et al. ^21^ | 2006 | M | 3 | Abdominal distention | US, CT | NA | OR | NA | NA |
| Wildhaber et al. ^22^ | 2006 | F | 1,5 | Pain, Vomiting | X-ray, US, CT | NA | LR | Complete | 18 |
|  |  | M | 4 | Pain | X-ray, US, CT | 12 | LR | Complete | 18 |
| Wilson et al. ^23^ | 2006 | F | 10 | Pain, abdominal distention | US, CT, MRI | 17 | OR | Complete | NA |
| Pratap et al. ^24^ | 2008 | M | 2 | Fever | US, CT | 8 | NA | Complete | NA |
| Singh et al. ^25^ | 2009 | F | 12 | Pain | US, MRI | 6 | LR | Complete | NA |
| Gümüştaş et al. ^26^ | 2013 | F | 8 | Pain, Nausea, Vomiting | US, CT | 20 | OR | Complete | NA |
| Present case | 2019 | M | 17 | Abdominal pain | US, CT | 14 | LR | Partial | 7 |

Abbreviations:

M: Male; F: Female; US: ultrasound, CT: computed tomography; MRI: Magnetic resonance imaging;

NA: not available; AS: aspiration and sclerotherapy; OR: Open resection; LR: Laparoscopic resection
